# Design and validation of a medical robotic device system to control two collaborative robots for ultrasound-guided needle insertions

**DOI:** 10.3389/frobt.2022.875845

**Published:** 2022-09-28

**Authors:** Johann Berger, Michael Unger, Johannes Keller, C. Martin Reich, Thomas Neumuth, Andreas Melzer

**Affiliations:** ^1^ Innovation Center Computer Assisted Surgery (ICCAS), University of Leipzig, Leipzig, Germany; ^2^ Institute for Medical Science and Technology (IMSaT), University Dundee, Dundee, Scotland

**Keywords:** surgical robotics, robotic biopsy, image-guided robot, collaborative robotics, IEEE 11073 SDC

## Abstract

The percutaneous biopsy is a critical intervention for diagnosis and staging in cancer therapy. Robotic systems can improve the efficiency and outcome of such procedures while alleviating stress for physicians and patients. However, the high complexity of operation and the limited possibilities for robotic integration in the operating room (OR) decrease user acceptance and the number of deployed robots. Collaborative systems and standardized device communication may provide approaches to overcome named problems. Derived from the IEEE 11073 SDC standard terminology of medical device systems, we designed and validated a medical robotic device system (MERODES) to access and control a collaborative setup of two KUKA robots for ultrasound-guided needle insertions. The system is based on a novel standard for service-oriented device connectivity and utilizes collaborative principles to enhance user experience. Implementing separated workflow applications allows for a flexible system setup and configuration. The system was validated in three separate test scenarios to measure accuracies for 1) co-registration, 2) needle target planning in a water bath and 3) in an abdominal phantom. The co-registration accuracy averaged 0.94 ± 0.42 mm. The positioning errors ranged from 0.86 ± 0.42 to 1.19 ± 0.70 mm in the water bath setup and from 1.69 ± 0.92 to 1.96 ± 0.86 mm in the phantom. The presented results serve as a proof-of-concept and add to the current state of the art to alleviate system deployment and fast configuration for percutaneous robotic interventions.

## 1 Introduction

Image-guided percutaneous biopsies provide the basis for diagnosis and staging in cancer therapy by sampling tumor tissue. Traditionally, a physician inserts a needle into the target organ (typically liver, kidney, breast, lymph nodes) based on pre-operative computed tomography (CT) or magnetic resonance imaging (MRI). The target deformations and needle deflections due to variations in tissue density often require intraoperative image guidance. Depending on the pathology and target location, CT and MRI offer high imaging quality and tissue differentiation also during interventions. However, the radiation exposure of CTs, the limited available space, and the long image acquisition times of MRIs often impede elementary biopsies. Interventional ultrasound (US) imaging provides more flexible image guidance, despite lower resolution (Roberts et al., 2020). Facing these constraints, robotic systems can improve the efficiency and outcome of percutaneous interventions while lowering the burden for the patient.

In the last 5 years, the development of robots with different actuation technologies (electric, pneumatic, cable-actuated, etc.) and degrees of freedom (2–7 DOF) provided high precision and repeatability for needle insertions (Siepel et al., 2021). These systems represent successfully deployed solutions, but despite their advantages, they are still not state-of-the-art in the surgical domain. This often affiliates to a lack of user acceptance, due to the high complexity of operation and the difficulties to integrate robotic systems into the operating room (OR). Besides a lack of full OR integration, the extensive costs limit the number of deployed robots even further. Today, only hospitals with maximum care can provide the infrastructure and resources to acquire modern robots. The restraint of individual systems to single or specific types of use-cases enhances this problem even more (Hoeckelmann et al., 2015; Schleer et al., 2019).

In industrial setups, similar challenges were discussed since the early 2010s, proposing an effective solution in the form of service-oriented architectures (SOA). By providing system functionalities as services to all relevant participants in a network, this approach was identified to not only achieve the ability for the integration of different systems but also improve reusability, scalability, and setup-time (Veiga et al., 2009; Cesetti et al., 2010; Oliveira et al., 2013; Cai et al., 2016). A variety of tools and frameworks to implement this approach in industrial settings has been published previously, e.g., the Robot Operating System (ROS^1^) or the Open Robot Control Software (OROCOS^2^). Although these tools can assist to overcome the highlighted problems, an effective utilization in medicine still lacks behind.

In 2001 Cleary and Nguyen discussed the necessity of flexible system architectures, for “[…] medical robotics to evolve as its own field […]” (Cleary and Nguyen, 2001). As a response, research was conducted to realize a standardized, vendor-independent medical device communication (Arney et al., 2014; Okamoto et al., 2018). Promising solutions were presented to improve interoperability in the operating room (OR) resulting in the approval of the IEEE 11073 SDC standard for service-oriented device connectivity (Kasparick et al., 2015, 2018; Rockstroh et al., 2017). This standardized approach already showed benefits, such as technical context-awareness to enable intelligent and cooperative behavior of medical devices (Franke et al., 2018). The SDC standard may also improve the modularity and interoperability of surgical robotic systems and, thereby, support the efforts towards wider adoption.

The principles of collaborative robots showed additional approaches in industrial setups to increase usability when sharing a workspace between humans and robots. The optimized fluency and synchronization of task transitions between the user and the assisting robot in collaborative setups may also yield high precision during percutaneous interventions while alleviating stress in the surgical team (Ajoudani et al., 2018; Hoffman, 2019).

In previous works, the application of novel collaborative approaches in robot-assisted US guided biopsies showed promising results (Berger et al., 2018a; Berger et al., 2018b). The implementation of a force-based touch gesture interaction in an experimental setup with a KUKA robot provided the basis for a preliminary user validation. The study involved nine participants of technical and/or medical background performing biopsies of two lesions in an abdominal phantom, both manually and with robotic assistance. The setup comprised a single robot moving an ultrasound device with an integrated guide for manual needle advancement and optical tracking tools for target planning. The users rated the intuitiveness and alleviation of needle guidance with touch gestures and hand-guided robot movement in a questionnaire, resulting in an overall positive evaluation of the interaction concept and the alleviation of the needle insertion tasks. In all performed biopsies, the target lesions were hit on the first try when using robot assistance.

To build upon these findings and to extend the preliminary system, this work explores the potential of two KUKA arms deployed in a collaborative setup for US guided needle insertions. It shall provide an addition to the current state of the art and prove the concept of a flexible robotic setup to support image-guided biopsies. The underlying control software incorporates multiple robots and medical devices to prove the possibility to deploy complex technical setups integrated into a standardized dynamic device network. It aims to promote the information exchange between commercially available collaborative systems in the operating room and to provide reusability in varying use cases. The introduction of collaborative interaction principles shall further increase acceptance when operating with robot assistance in percutaneous interventions.

## 2 Materials and methods

The main goal of this work was the implementation of a robot control system that utilizes the advantages of the SDC standard and collaborative interaction principles for robot-assisted needle insertions. It should incorporate existing robotic systems, to improve reusability and flexibility. The targeted robots must, therefore, support control and information exchange *via* SDC.

### 2.1 Robots as SDC medical devices

The SDC standard implements a service provider and consumer architecture with medical devices acting as providers, consumers, or both. Providers publish medical device capabilities and consumers interact as needed by either request and response or *via* subscription. The communication between provider and consumer supports dynamic device discovery with unique device identifiers (UDI) and is realized *via* a standard Ethernet connection. In the SDC standard family, a medical device information base (MDIB) represents the device as a pair of medical device description (MdDescription) and medical device state (MdState). The MdDescription contains the supported conditions and variables stored as metrics of distinct types (e.g., numeric, string, enumeration). Metrics are grouped into channels to categorize the device capabilities, which in turn are assigned to a virtual medical device (VMD). Multiple VMDs can be further combined into a single medical device system (MDS). All VMDs, channels, and metrics are identifiable by unique handles. The MdState contains the actual values of available metrics and device characteristics. A consumer can remotely control a provider (and thereby the medical device) *via* a service and control object (SCO) which defines set operations on the metrics or activation operations to trigger specific behavior. (Kasparick et al., 2015, Kasparick et al.,2018) describe the structure of SDC in more detail.

**FIGURE 1:**
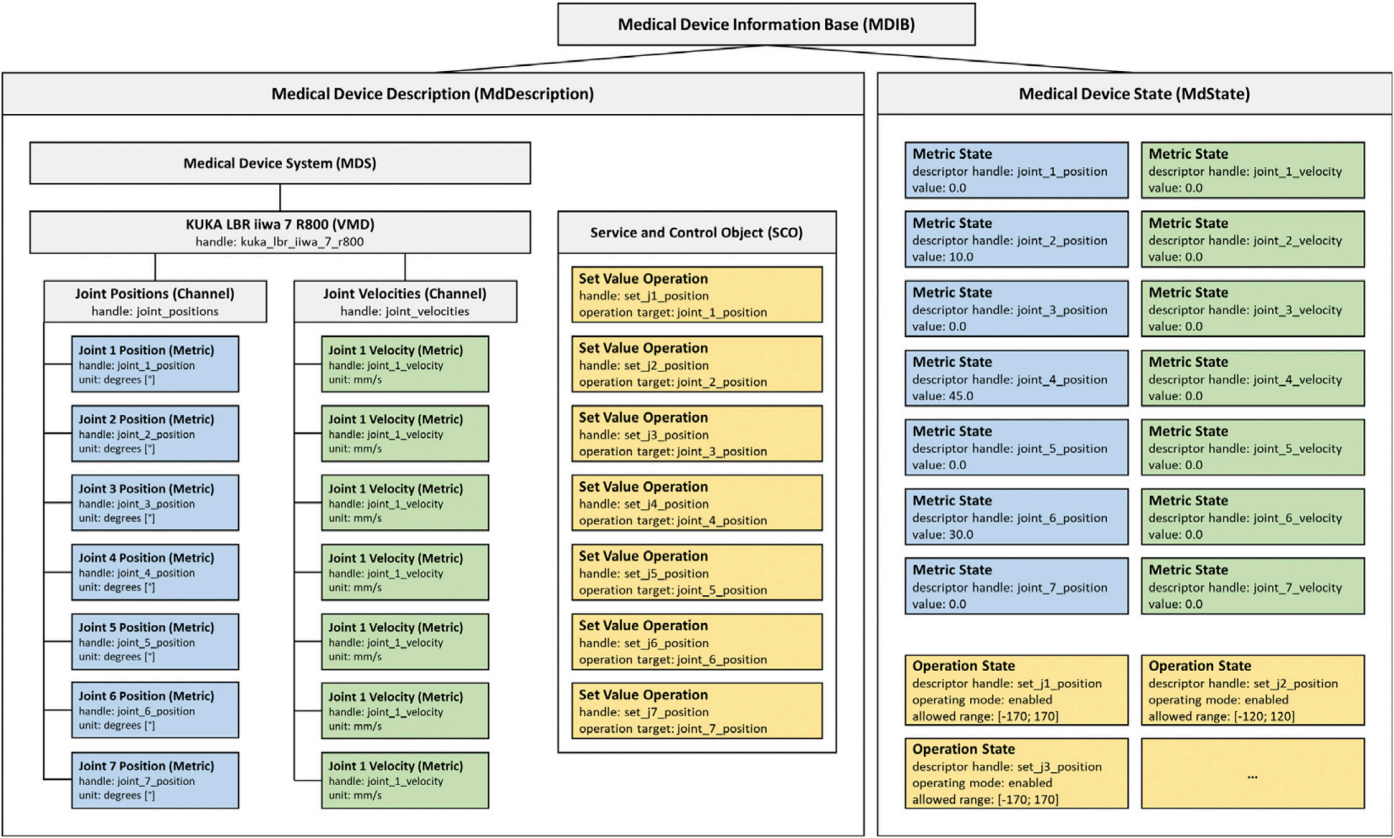
Example MDIB for a KUKA LBR iiwa 7 R800. The MdDescription includes the KUKA VMD with joint positions (blue) and velocities (green). The SCO provides operations to set position values (yellow) and the MdState contains exemplary values and ranges for the metrics and operations.

The MDIB structure allows for the representation of all essential characteristics of any robotic device, like the available degrees of freedom, their current position, velocities, etc. For example, the possibility to implement a KUKA LBR iiwa 7 R800 as an SDC medical device was shown before (Berger et al., 2019). Figure 1 visualizes a possible MDIB with joint positions and velocities.

### 2.2 MERODES: The medical robotic device system

To exploit the features of SDC as best as possible, this work presents a flexible software framework with interchangeable manipulators and configurations for deployment in different use-cases. The following sections describe the design of this Medical Robotic Device System (MERODES) based on the SDC MDIB architecture and its deployment with two robotic arms for US-guided biopsies.

#### 2.2.1 System design

The core component of MERODES is a central MDS that manages all involved robotic devices represented by their respective VMD. This enables the system to dynamically include or exclude any proprietary robot on initialization (if an SDC conform VMD is available). Depending on the use case, the system hides or publishes the respective services of each included robot in the network.

Operating multiple robotic devices requires a shared coordinate space. MERODES additionally acts as a consumer of SDC compatible tracking devices and, thereby, supports the usage of surgical tracked navigation principles. The active tracking device can be selected on initialization. The system includes an additional comprehensive VMD that provides services and metrics for the overall system control (e.g., the selected tracking device, activation commands for starting the registration process, transformations between the robot coordinate systems).

**FIGURE 2:**
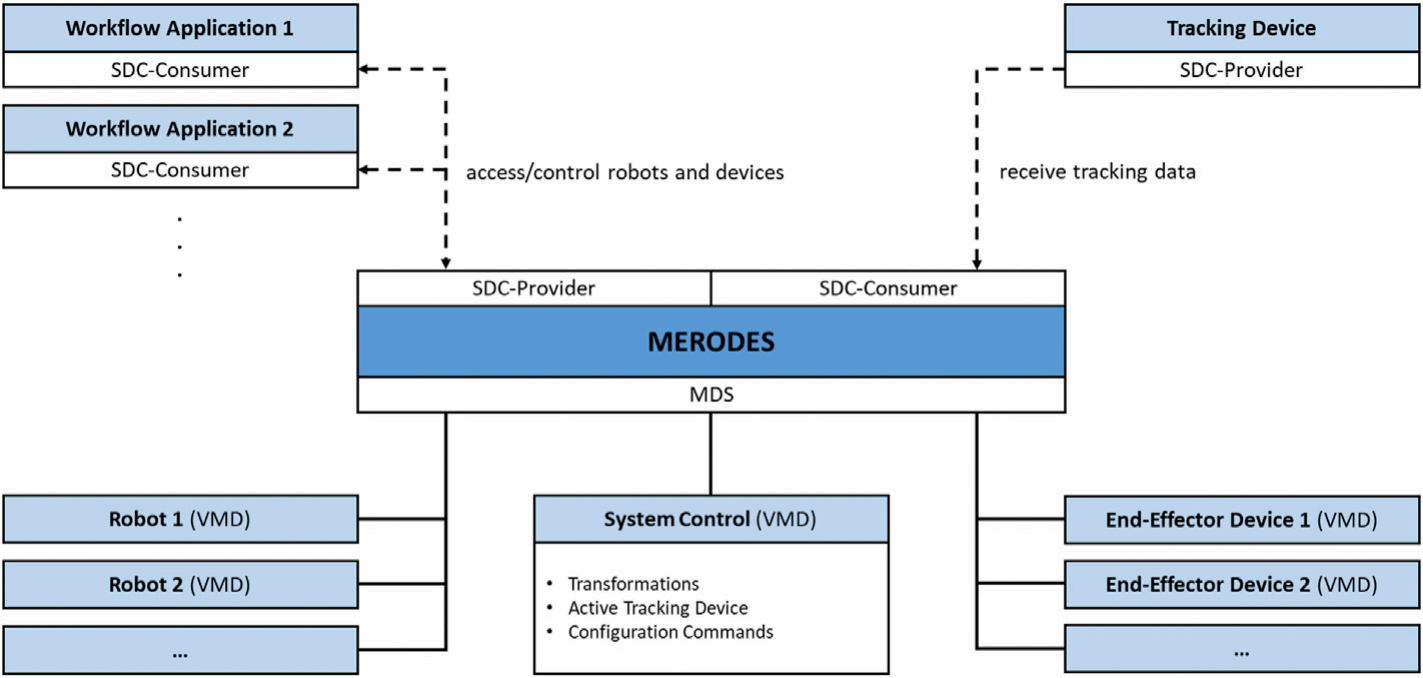
Overview of the MERODES architecture.

Many robotic systems support interchangeable end-effectors, or are solely built to control proprietary tools (e.g., KUKA). These end-effectors may comprise medical devices themselves, and can be included in the MDS if their services are required in the network (e.g., starting/stopping diagnostic or therapeutic functions). All remote capabilities of the included devices and robots are stored in the SCO when adding a VMD to the system. MERODES separates the workflow logic from the incorporated robots. Depending on the intervention, use case-specific workflow applications act as consumers, access the robot services, and implement the robotic behavior and user interactions. This allows for a flexible setup by changing the consumer application to use the same robots for different tasks and vice versa. Figure 2 shows the MERODES architecture.

#### 2.2.2 The collaborative robot setup

**FIGURE 3:**
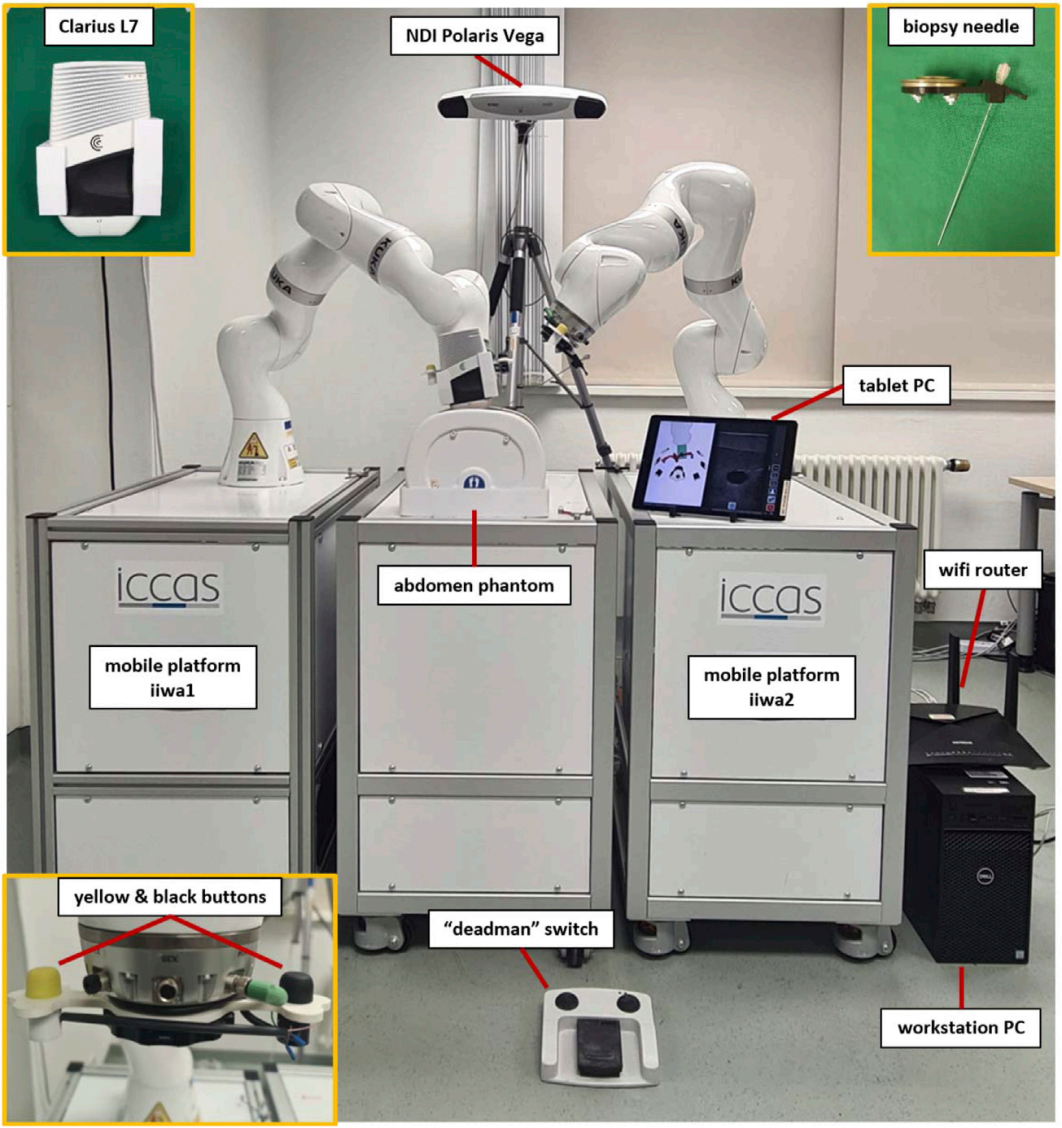
The experimental dual-arm setup with two KUKA LBR iiwa 7 R800 robots mounted on mobile platforms. The manipulators are positioned at an abdominal phantom and the tablet PC displays a workflow application for US-guided biopsies (described in more detail in Section 2.2.3).

To validate the feasibility of deploying MERODES for US-guided needle insertion, the robotic setup in this work comprised two KUKA LBR iiwa 7 R800 robotic arms (KUKA AG, Germany). Both robots and their respective cabinet PCs were mounted on custom-built mobile platforms. The biopsy workflow was based on one robot (iiwa1) providing US image guidance and target positions for the second robot (iiwa2) steering the needle (see Section 2.2.3). A Clarius L7 (Clarius Mobile Health Corp., Canada) served as the diagnostic US device, attached to the flange of iiwa1 *via* a 3D printed mount. An US compatible biopsy needle (length 180 mm) was similarly mounted to iiwa2. An NDI Polaris Vega (Northern Digital Inc., Canada) acted as SDC provider and enabled the utilization of both robots in a shared coordinate space by co-registration. Both robot platforms comprised rigid markers for optical tracking, attached at the back of the setup. The markers were not integrated with the end-effectors to avoid line-of-sight problems when working in human-robot-collaboration and to allow for the flexible exchange of tools without worrying about the tracking device. This approach allowed for changes in the deployment of both arms after initial registration. A central workstation PC (DELL Precision 3630 Intel (R) Core™ i7-8700K CPU @ 3.70 GHz, 32.0 GB RAM) served as the control unit to execute the MERODES software and other dependencies. The collaborative nature of the KUKA robots allows for the utilization of human-machine-interaction principles, like shared target manipulation and fluent changes between automated and manual tasks (Hoffman, 2019). Both robots, therefore, were augmented with two interactive buttons (black and yellow) installed between the flanges and the respective tools to perform hand guiding operations or other workflow-dependent actions. The electric flange interfaces of the KUKA robots directly routed the button input signals through the manipulators to a USB port of the workstation. A separate tablet (iPad Pro model A1652) allowed for remote access to the system while working in collaboration with the robots. All devices and computers were connected to a smart wifi router (NETGEAR nighthawk ×10 ad7200) to form a closed network. Figure 3 provides an overview of the experimental dual-arm setup (DAS).

**FIGURE 4:**
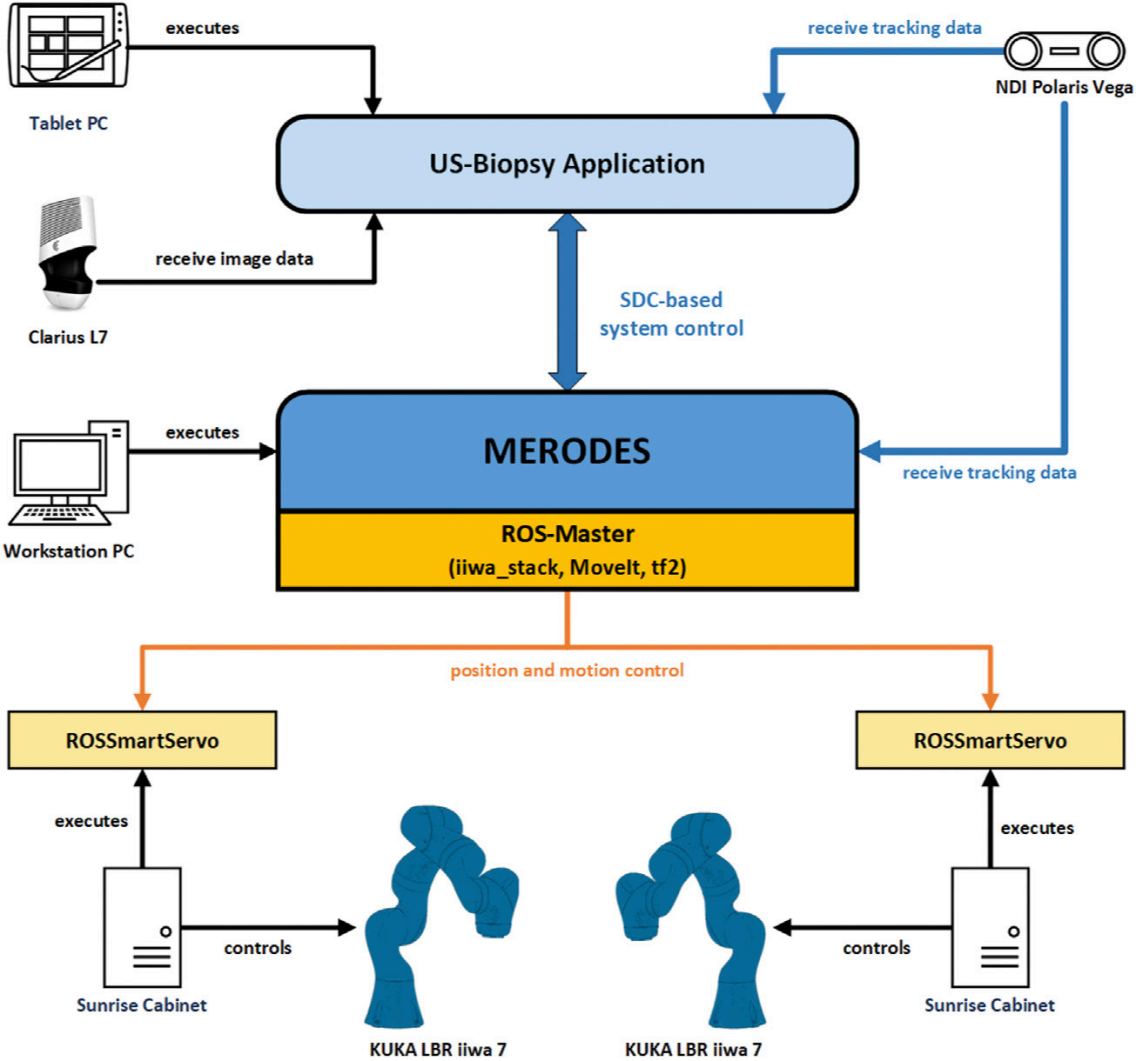
The communication and information exchange between the components of the dual-arm setup. Blue arrows represent SDC-based communication; orange arrows represent ROS-based communication.

The software in the DAS incorporated a shared planning environment for both robots using the MoveIt framework and ROS. In this instance, MERODES accessed and controlled the robots using the iiwa_stack application^3^ (Hennersperger et al., 2017) and translated between ROS inherent topics and SDC messages. The iiwa_stack application was extended to include multiple robots rooted in the coordinate system of the utilized tracking device, allowing for path planning with collision avoidance between the robots, the patient, and other peripherals. The tf2 library^4^ provided the transformations between both robots and the tracking space. On initialization, MERODES dynamically mirrored all available ROS services and generated the associated VMDs for each robot. Hence, the DAS encompassed two VMDs for iiwa1 and iiwa2, respectively. Besides the ROS services, additional metrics for the attached buttons at the robot flanges were included in the robot VMDs to publish the interactive states (pressed/released) to the SDC network. Based on previous works, the possibility to access applied forces on the manipulators allowed for the mapping of touch gestures on the end-effectors to specific commands (Berger et al., 2018b). The system published the received gestures (e.g., left push, right push, etc.) to the SDC network, as well. MERODES and all ROS dependencies were implemented in C++ and compiled/executed on a virtual machine (OracleVM, version 5.2.26) running Ubuntu 16.04. Since ROS and SDC are not real-time capable, the iiwa_stack inherent ROSSmartServo application managed all real-time dependent motion controls on the KUKA Cabinets. To provide additional safety, a foot pedal (directly connected to the Cabinets) served as a “deadman switch” to enable/disable robot movement. A consumer application for US-guided biopsies (written in C++ with Qt5^5^ and built for Windows 10 with the Visual Studio 2019 toolset) provided a graphical user interface (GUI) that was streamed to the tablet. It managed the configuration and control of the robotic system *via* SDC (see Section 2.2.3). All SDC dependencies were implemented using the SDCLib/C^6^ library. The DAS components are visualized in Figure 4.

#### 2.2.3 Ultrasound-guided biopsy application

The workflow for needle insertion incorporated treatment planning in US images and switching between motion-control modes of the manipulators (i.e., hand guidance and automated positioning). On initial connection to MERODES, the consumer application configured the system settings to assign the tool center points (tcp) for each robot (iiwa1—Clarius tcp, iiwa2—needle tcp). The button signals (sent *via* SDC while pressing the buttons) were mapped to activate different hand guiding modes as follows:

• Translation hand guidance (iiwa1 and iiwa2—yellow buttons): The end-effector can move only in translational DOF. Any rotational movement of the tcp is constrained.
• Rotation hand guidance (iiwa1—black button): The end-effector can move only in rotational DOF. Any translational movement of the tcp is constrained.
• Trajectory hand guidance (iiwa2—black button): The end-effector can move only along the trajectory axis (i.e., the effective direction of the tool). Any translation along the orthogonal axes and any rotation of the tcp is constrained.

**FIGURE 5:**
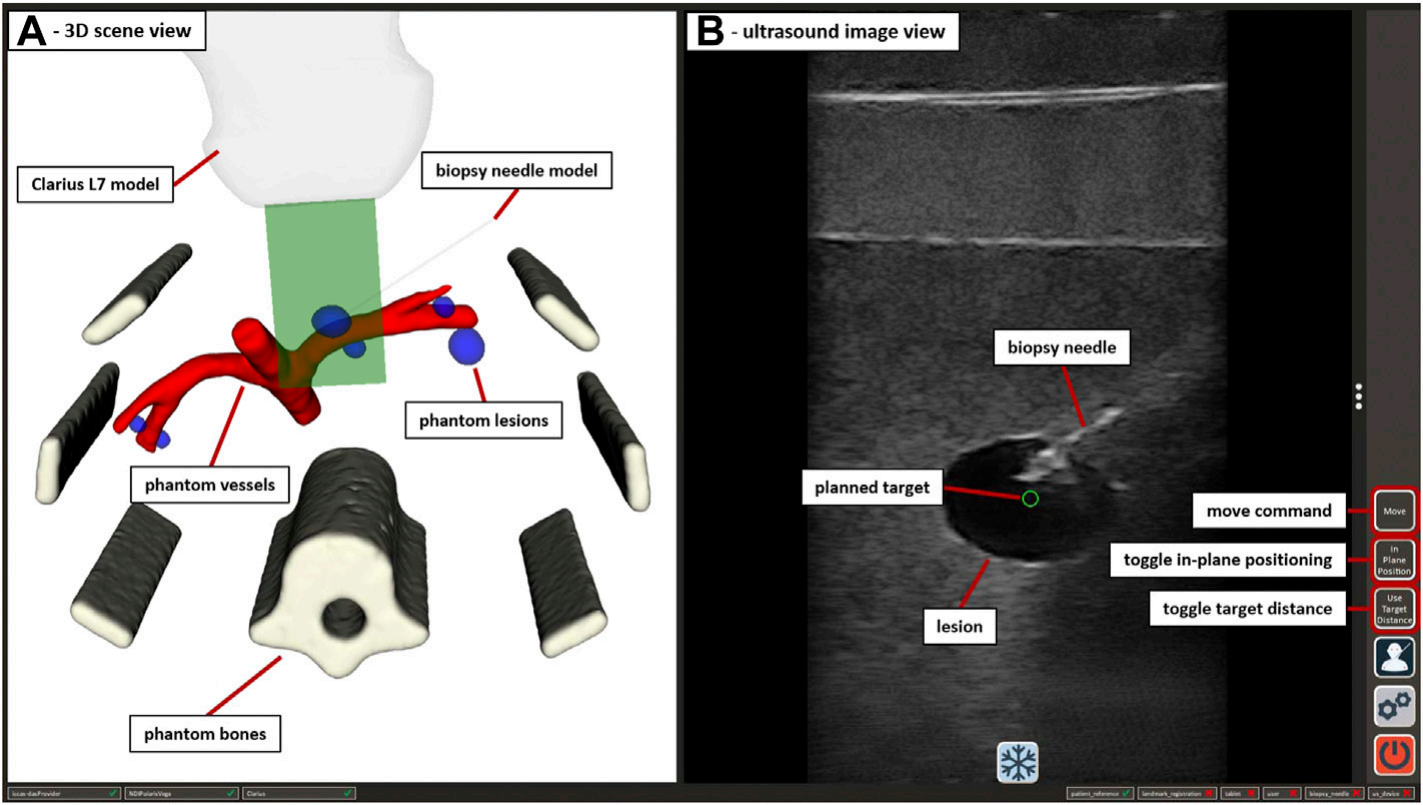
The GUI of the US-guided biopsy application. **(A)** displays the 3D scene view containing the segmented CT/MRI data of the abdominal phantom used in Section 2.3.3 and 3D representations of the used end-effectors. **(B)** shows the US image view with the planned target position (green circle) and the image of an inserted biopsy needle. Interactive GUI elements for movement commands and switching between positioning modes are listed in a toolbar at the right border.

The application received 2D image data from the US device *via* the Clarius Cast API^7^ and displayed it in an interactive view, as shown in Figure 5. The treatment planning was performed on the tablet PC by clicking/touching on the target position for the needle tip inside the US image. Using the transformations of the System Control VMD (see Figure 2) the 2D click position was represented as the corresponding 3D position in the coordinate space of iiwa2. The application supported two options for the orientation (rotation around the tcp) of the needle at the target point: perpendicular to the imaging plane or parallel to the imaging plane with a changeable angle for the in-plane position (see Section 2.3.2). Sending the target position and rotation of the needle to MERODES *via* SDC, the system calculated the robot path from the current position to the target using MoveIt and returned a success message if the target was reachable. Reachable targets were represented by a green circle at the click position in the US image. A button in the GUI and/or a right push gesture on the needle manipulator enabled the activation of automated movement to the target. The insertion of the needle is the most critical part of the procedure. To keep the clinical personnel in charge of all invasive tasks, the option to position the needle at a non-invasive distance to the target, subsequently called pre-position, was included. Executing the path planning with this condition the pre-position was set by shifting the user-selected target position 200 mm away from the target along the needle trajectory. Subsequently, the user could move iiwa2 with trajectory hand guidance to advance the needle manually while staying on target. For better orientation, a separate scene view provided the option to visualize CT and MRI-based 3D patient models and the robotic end-effectors (see Figure 5). The application was connected to the tracking device, to perform patient registration and to include additional optical tools (e.g., pointers) for planning purposes, if so desired.

Using the US-guided biopsy application, the needle insertion workflow involved the following three steps:

1. Locating the target structures (e.g., tumor tissue) with US imaging, using translation and/or rotation hand guidance with iiwa1 while orienting in the 3D scene
2. Planning the target needle position in the US image and performing automated movement of iiwa2 to the pre-position
3. Advance the needle under US image monitoring, using trajectory hand guidance with iiwa2 until the needle is visible in the target position

The design of MERODES and the depicted application intends to allow the interventional radiologist and/or surgeon to perform all tasks independently.

### 2.3 System validation

The validation of MERODES and the biopsy workflow application included three test sets; 1) Measuring the co-registration accuracy of both robots and the referring rigid markers with an optical tracking tool 2) Assessing the accuracy of positioning iiwa2 depending on the US-guided targeting provided by iiwa1 in water bath 3) Performing the biopsy workflow and measuring the accuracy on an abdominal phantom.

#### 2.3.1 Robot co-registration

**FIGURE 6:**
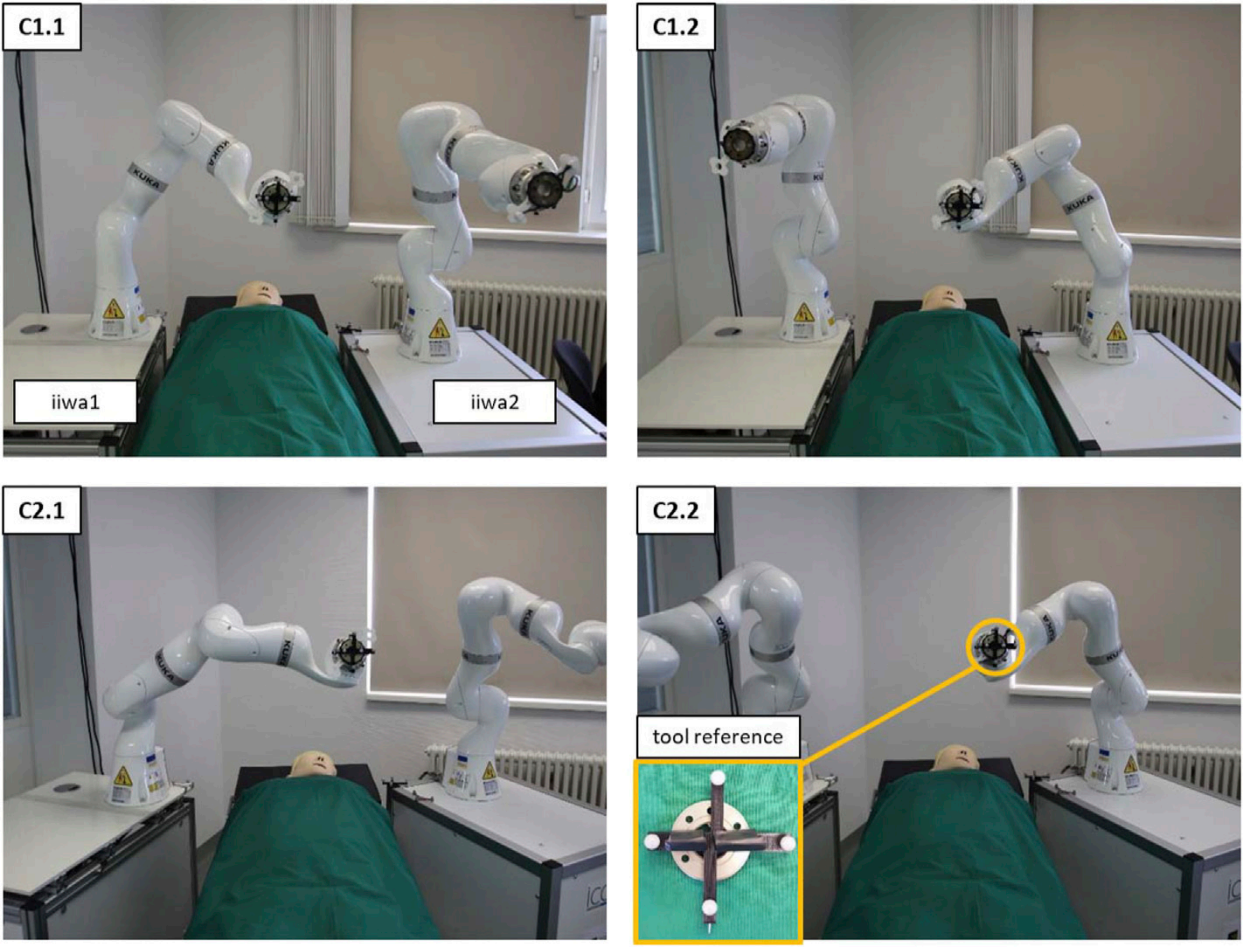
The setup of both robot platforms at an OR-table to measure the interdependent positioning accuracy. C1 shows the configuration in parallel to the table; C2 shows the angled configuration. C1.1 and C2.1 show examples of the 9 initial positions of iiwa1. C1.2 and C2.2 depict the corresponding positions of iiwa2 after solving the coordinate transformation. The pictures show the setup from the visual perspective of the tracking camera.

To achieve reliable accuracy measurements, a custom-made tool provided the reference end-effector for the interdependent positioning of both robots. As shown in Figure 6, the tool comprised a 3D cross shape, mounting four infrared marker spheres. An integrated nail tip served as the tcp. The marker spheres were calibrated with the NDI Polaris Vega to compose an optical tool reference (TR). The TR was attached to an FWS flat change system (SCHUNK GmbH & Co. KG, Germany) for a fast exchange between the robots and added as an end-effector to the robot arms toolset. MERODES supports landmark registration functionality, which is common in tracked surgical navigation. This method was used to determine the transformations between the coordinate spaces of the robots and their respective rigid markers, by moving the TR to five distinct positions (visible by the tracking camera) and recording the coordinates in both robot and tracking space. The resulting transformations were added to the tf2 tree of MERODES, enabling the transformation between both robot coordinate systems.

The accuracy measurements of the co-registration involved the movement of the robots to nine distinct positions in three repetitions. First, iiwa1 steered to the predefined positions to record the coordinates of the TR in tracking space (using the Vega camera) and in robot space (using the Cartesian position of the manipulator). Subsequently, the tool was mounted to iiwa2, to move to the same positions, by solving the coordinate transformation from iiwa1 space to iiwa2 space. The tracking camera similarly recorded the coordinates of the TR for iiwa2 to calculate the Euclidean distance between the corresponding positions. The procedure was conducted for two deployment configurations of the robot platforms (C1—deploying the robots in parallel at an OR-table; C2—deploying the robots in an angled position). Figure 6 depicts the two configurations and the interdependent positioning.

#### 2.3.2 Ultrasound-image target planning

The system must provide sufficient accuracy, not only when directly transforming between the tcp coordinates, but also for target positions derived from US images. To measure the error introduced into the system by translating the clicked coordinates in the image view to a target position in robot coordinates, an additional validation was performed using a water bath. Performing the tests in water ensured moving the needle without introducing errors by deformation, e.g., due to deflections while advancing it through tissue (as addressed in Section 2.3.3). Consequently, the data resulting from this setup will serve as a reference for improving the system quality in future optimization steps.

**FIGURE 7:**
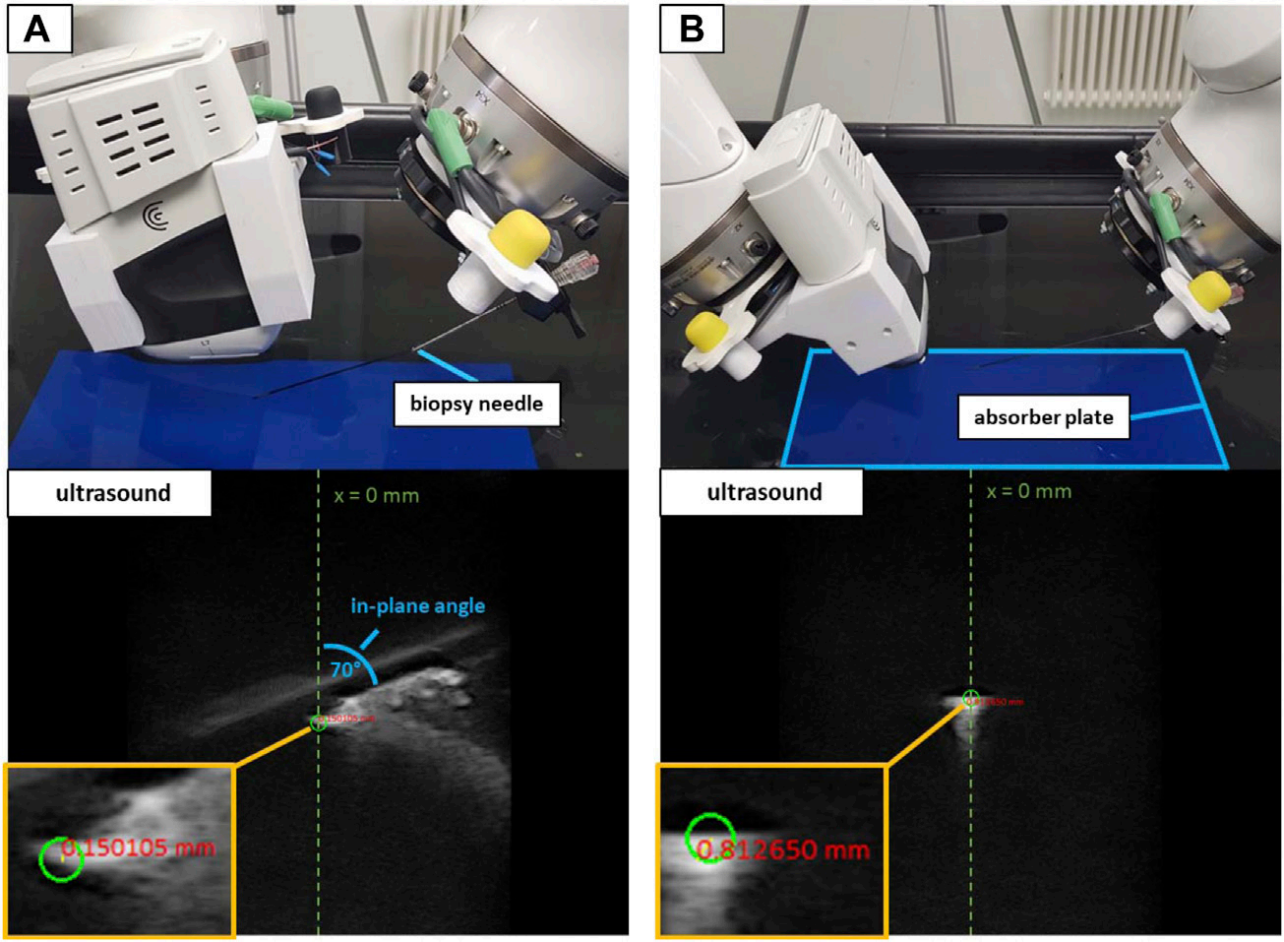
The water bath setup with in-plane positioning at 70° **(A)** and orthogonal positioning **(B)**. White artifacts in the US images depict the reflections of the needle. The green circles show the planned target at x = 0 mm and a depth = 40 mm. The red numbers show the distance from the planned target to the approximated center of the needle tip in the US image.

The imaging array of the Clarius device was submerged in water and directed down onto an US absorbent plate (Aptflex F28^8^) to minimize US reflections. The device was slightly angled to the water surface, allowing for better freedom of movement for the second robot. At first instance, the validation involved automated positioning, equivalent to the co-registration measurements in Section 2.3.1. Pre-defined 2D coordinates laid out as a 5 × 4 grid in the imaging plane served as the target positions. The 20 coordinates comprised values of −10, −5, 0, 5, and 10 mm horizontally (x-axis, with x = 0 being the horizontal center of the image) and 20, 40, 60, and 80 mm of depth (y-axis, with y = 0 being the upper border of the image). The 2D coordinates (given in relation to the tcp of iiwa1) were translated into 3D and iiwa2 placed the needle tip at the resulting target positions, by applying the robot-to-robot transformation. Positioning the US compatible biopsy needle inside the imaging plane provided a sharp contrast, enabling the visual assessment of reaching the planned targets. The targeting error was assessed manually in the US image, by measuring the distance between the planned 2D coordinates (green circle) and the approximated positions of the needle tip. Since only 2D imaging was available and to gain information for the missing dimension, the procedure was performed placing the needle orthogonally to the imaging plane and in parallel to the imaging plane at an angle of 70°. Figure 7 visualizes both the orthogonal and in-plane positioning with the resulting US images for the x-/y-coordinate of 0/40 mm.

**FIGURE 8:**
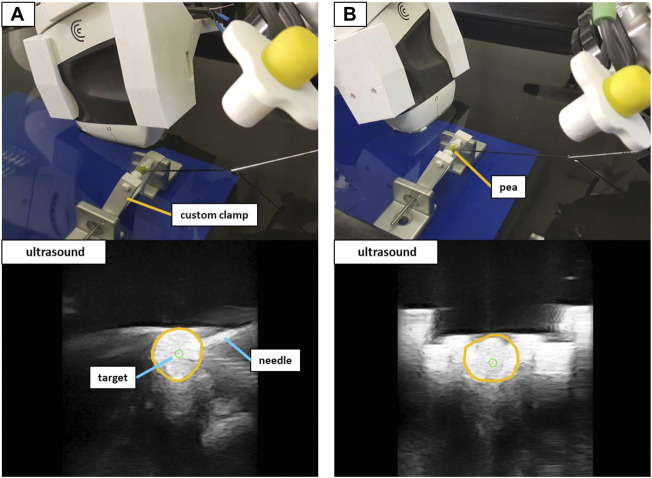
The experimental setup to target peas in the water bath. Shown are the in-plane positioning **(A)** and orthogonal positioning **(B)** of the US device. The orange circles in the US images outline the peas.

To validate the possibility to puncture sufficiently small structures when using the trajectory hand guidance, peas (∼6 mm in diameter) provided the targets for a second water bath validation as shown in Figure 8. A 3D-printed custom clamp held the submerged peas in place to perform needle insertions. The clamp could be rotated to adjust the height of the peas and was placed at five arbitrary positions on the absorber plate in the water bath. iiwa1 positioned the US device to visualize the center of the peas for both orthogonal and in-plane targeting. Clicking inside the area of the peas in the US image provided the target positions. The movement planning for iiwa2 positioned the robot at distance to the targets (as described in Section 2.2.3) and the needle was advanced *via* hand guidance until perforating the peas. The tip of the needle was not visible within the peas (see Figure 8). Therefore, instead of measuring the positioning error to the planned target, a binary analysis was performed (the target was perforated or not perforated).

#### 2.3.3 Phantom validation

**FIGURE 9:**
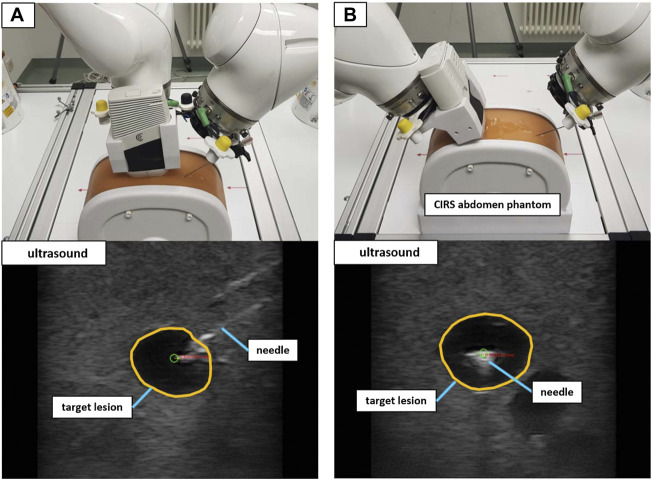
The validation setup to puncture lesions inside an abdominal phantom with trajectory hand guidance. Shown are the in-plane positioning **(A)** and orthogonal positioning **(B)** for the same target lesion. The green circle marks the planned needle tip position.

A validation setup with a triple modality 3D abdominal phantom^9^ (CIRS Inc., United States) allowed for a performance test that was closer to reality. Abiding by the needle insertion workflow described in Section 2.2.3, 40 needle insertions were performed on two distinct lesions inside the phantom. The first lesion (L1) was located at a depth to the surface of ∼42 mm with a size of ∼12 mm in diameter. Lesion 2 (L2) resided at a depth of ∼35 mm with a size of ∼9 mm in diameter. Again, the needle orientation was planned for both in-plane and orthogonal positioning (20 insertions for each orientation). Figure 9 illustrates the phantom setup with the corresponding US images for L1.

**FIGURE 10:**
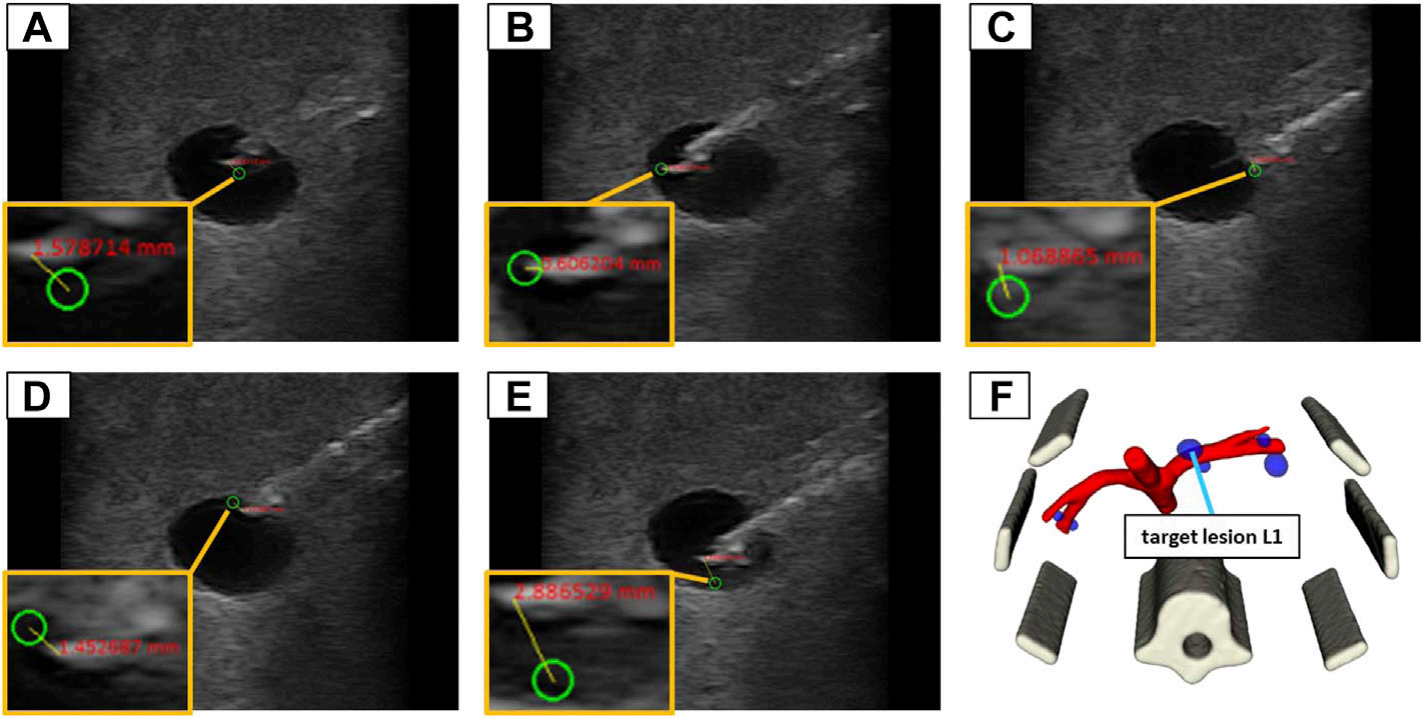
The 5 target positions inside of a lesion (L1) in the abdominal phantom for an in-plane needle positioning: **(A)** in the center; **(B)** left border; **(C)** right border; **(D)** top border; **(E)** bottom border; **(F)** illustrates the 3D model of the phantom to provide an overview where the lesion is located in 3D space.

Similar to the validation in water, the needle images allowed for the manual assessment of the position error, by measuring the 2D distance between the needle tip and the planned target. The needle insertions were conducted in eight iterations (four in-plane and four orthogonal). Each iteration comprised the placement of the US device to visualize the target structure and the planning of five target positions inside the lesion (at the left, right, top, and bottom borders as well as in the center) as depicted in Figure 10. The needle was advanced in hand guidance with the goal to minimize the distance of the needle tip to the planned target. The insertion depth was determined, relying on the visual feedback in the US image view.

## 3 Results

The results of this work represent the performance of MERODES when controlled by an SDC consumer application for US-guided biopsies. All workflow-related tasks described in previous sections were performed solely by exchanging SDC messages and commands between the biopsy application and the dual-arm setup. The accuracy measurements of the three validation test sets yielded the following results.

### 3.1 Co-registration accuracy

The mean error of the interdependent positioning for deployment configuration C1 was 0.93 ± 0.29 mm. Deployment configuration C2 resulted in a mean target deviation of 0.95 ± 0.38 mm. The overall positioning error of the dual-arm setup on average was 0.94 ± 0.42 mm. The measured mean errors and standard deviations for all 9 positions in both configurations are listed in Table 1.

**TABLE 1:** The mean position errors and standard deviations for all 9 positions in the co-registration setup for the deployment configuration C1 and C2. All values are provided in mm.

Position	C1		C2	
	Error [mm]		Error [mm]	
	Mean	Std	Mean	Std
1	1.14	0.03	0.78	0.24
2	0.89	0.61	1.02	0.38
3	1.00	0.30	1.29	0.38
4	1.08	0.34	0.49	0.10
5	0.62	0.14	0.71	0.30
6	0.56	0.33	0.79	0.50
7	1.04	0.22	0.85	0.40
8	1.14	0.22	1.83	0.51
9	0.62	0.43	0.71	0.31

### 3.2 Targeting accuracy in water bath

When positioning the Clarius device in a water bath, *iiwa2* reached all pre-defined 2D targets, utilizing automated movement directly to the target positions. Measuring the target distances in the US images using in-plane positioning resulted in a mean 2D error of 0.86 ± 0.42 mm. The mean 2D positioning error in orthogonal needle placement was 1.19 ± 0.70 mm. Table 2 contains all target coordinates in the image (x, y) and the corresponding measured distance to the needle tip for in-plane and orthogonal positioning.

**TABLE 2:** The 2D distance measurements for in-plane and orthogonal positioning in the water bath setup. The click positions of the planned targets are provided as x- and y-coordinates in mm.

In-plane positioning			Orthogonal positioning		
x [mm]	y [mm]	Distance [mm]	x [mm]	y [mm]	Distance [mm]
−10.0	20.0	0.69	−10.0	20.0	0.62
−10.0	40.0	0.24	−10.0	40.0	0.71
−10.0	60.0	0.94	−10.0	60.0	1.57
−10.0	80.0	1.65	−10.0	80.0	1.95
−5.0	20.0	1.00	−5.0	20.0	0.59
−5.0	40.0	0.11	−5.0	40.0	0.85
−5.0	60.0	0.71	−5.0	60.0	1.59
−5.0	80.0	1.35	−5.0	80.0	2.26
0.0	20.0	0.98	0.0	20.0	0.11
0.0	40.0	0.15	0.0	40.0	0.81
0.0	60.0	0.77	0.0	60.0	1.55
0.0	80.0	1.21	0.0	80.0	2.21
5.0	20.0	1.22	5.0	20.0	0.59
5.0	40.0	0.38	5.0	40.0	0.74
5.0	60.0	0.67	5.0	60.0	1.52
5.0	80.0	1.07	5.0	80.0	2.10
10.0	20.0	1.02	10.0	20.0	0.27
10.0	40.0	0.31	10.0	40.0	0.71
10.0	60.0	0.53	10.0	60.0	1.55
10.0	80.0	1.06	10.0	80.0	2.19

Advancing the needle with trajectory hand guidance was sufficiently accurate to target peas in water. At all five locations, the peas were punctured by the needle using in-plane and orthogonal positioning.

### 3.3 Targeting accuracy in phantom

The needle advancement in tissue resulted in higher errors. In-plane positioning yielded a mean 2D target deviation of 1.96 ± 0.86 mm. The orthogonal approach resulted in a mean 2D positioning error of 1.69 ± 0.92 mm. Planning positions at the borders of the lesions lead to missing the target volume in 8 out of 32 cases. Aiming at the lesion centers always resulted in a hit. The measured positioning errors for lesion 1 (L1) and lesion 2 (L2) are listed in Table 3.

**TABLE 3:** The 2D positioning errors for in-plane and orthogonal positioning for lesions L1 and L2 in the abdominal phantom. The click positions of the planned targets are provided as x and y coordinates within the US image. A binary assessment of hitting/missing the lesion is included.

	In-plane positioning				Orthogonal positioning			
	x [mm]	y [mm]	Distance [mm]	Lesion hit	x [mm]	y [mm]	Distance [mm]	Target hit
L1	2.16	46.45	0.84	Yes	−3.01	53.01	1.51	Yes
	−3.36	46.63	0.97	Yes	−9.22	53.13	1.49	Yes
	7.98	46.99	2.38	Yes	2.83	52.77	1.47	Yes
	2.28	42.25	1.65	No	−2.59	47.77	2.01	Yes
	2.10	51.37	3.30	Yes	−3.19	58.62	3.38	Yes
	0.42	45.18	1.58	Yes	−1.57	51.63	0.59	Yes
	−6.03	44.94	0.61	Yes	−7.23	51.57	1.81	No
	6.21	45.24	1.07	Yes	4.28	51.09	1.57	Yes
	−0.18	40.60	1.45	Yes	−0.96	47.05	3.12	Yes
	0.00	50.07	2.89	Yes	−1.75	56.45	2.82	Yes
L2	−3.06	38.96	1.67	Yes	2.11	45.24	1.41	Yes
	−6.83	38.84	2.26	No	−1.39	45.18	1.16	Yes
	0.12	38.84	2.50	Yes	5.67	45.12	1.00	Yes
	−3.36	35.42	1.07	No	1.99	42.40	1.09	No
	−3.00	42.37	2.63	Yes	1.99	48.86	4.32	Yes
	0.90	39.43	2.39	Yes	1.67	47.35	1.87	Yes
	−2.88	39.49	3.76	No	−1.27	47.53	2.17	Yes
	4.26	39.73	2.25	Yes	5.36	47.59	2.24	Yes
	0.72	36.07	2.40	No	1.75	44.22	0.90	No
	1.02	43.03	1.17	Yes	1.75	50.78	1.81	Yes

## 4 Discussion

This work presents the design and implementation of a collaborative robotic setup for US-guided needle insertions. The systems control software allows for the utilization of two KUKA lbr iiwa 7 R800 robotic arms in a flexible deployment. In the described setup, the robots control an US-imaging device and an US-compatible biopsy needle to perform interdependent target planning and positioning. The utilized hand guidance control and touch-gesture inputs support fluent transitions between automated movements and hands-on interaction. By utilizing encapsulated applications to separate the workflow logic from the incorporated robots, a quick configuration of the system depending on the clinical environment or user preferences can be enabled. The standardized integration *via* SDC additionally allows for variable setups to exchange information with peripheral medical devices (e.g., tracking cameras or ultrasound imaging).

To provide a performance validation, the system accuracy was measured while being controlled by the US-guided biopsy application. The system exhibited stable behavior at all times performing the interventional tasks under SDC based workflow control. The presented accuracies of 1.96 ± 0.86 mm (in-plane) and 1.69 ± 0.92 mm (orthogonal) in the phantom setup are within the range of currently available biopsy robots (Siepel et al., 2021). Since only 2D US-imaging was available in the described setup, the positioning errors could also only be measured in 2D. Due to the missing dimension, the 3D positioning error is expected to be higher. However, the same limitations apply for hand-guided needle positioning, in which the user would assess if a target was hit in a comparable way. The system can, therefore, be rated as non-inferior to the current state of the art concerning accuracy. The needle insertion was accomplished using a trajectory hand guidance mode in which the robot only allows for a single degree of freedom during the motion. Ideally, the needle should exactly follow the trajectory to the target structure. The targeting of peas in water promises sufficient accuracy for targets of ∼6 mm size. However, hand guidance with the KUKA robots relies on an impedance mode that increases the stiffness of the robot with an increasing deviation of the *tcp* from the specified path, i.e., movement orthogonally to the needle trajectory. The maximum achieved stiffness is limited and, therefore, introduces inaccuracies when moving through tissue. Further inaccuracies in reaching the desired target are introduced by the needle itself. Due to variations in tissue density, the needle deflects and bends during perforation. The described system does not provide needle steering. Although the positioning error can be detected using real-time US imaging, the error is not corrected. The binary assessment of hitting and missing targets in the phantom reflects this circumstance. In 8 out of 32 cases, the needle missed the targeted lesion due to additional deflections towards the surface of the phantom (negative y-direction in relation to the US image). Although lesion L1 was only missed in 2 cases when aiming for the upper or left borders, the deflections were especially problematic for the smaller lesion L2 (∼9 mm), for which most misses occurred. Active needle path correction plays an essential role in precise targeting and recent works, e.g., closed-loop deflection compensation as presented by Wartenberg et al. (2018) provide promising solutions. The capabilities of the proposed system should be enhanced by introducing a similar needle steering mode in the controlling workflow application to enable more precise targeting.

Positioning an US probe and a biopsy needle as described in this work is limited by the available space around the patient and the available degrees of freedom of the used robots. When using hand guidance, both robots have to operate very close to each other, as can be seen in Figure 9A. Reaching the desired target structure without robot collision is impeded by the target depth and the placement of the US device on the patient. The target planning with in-plane or orthogonal positioning introduces further restrictions to the freedom of movement. With the proposed workflow, the robot setup reliably reaches a needle depth of up to 60 mm (perpendicular to the skin surface) depending on the insertion angle, which is clinically acceptable for most percutaneous biopsies. Allowing the planning of needle positions independent of the imaging plane should be further investigated to reduce restrictions on the freedom of movement.

This work aims to reduce the complexity in the OR and increase acceptance of robotic interventions. The depicted system configuration introduces additional overhead that can impede reaching these goals, especially for relatively straightforward procedures such as biopsies. In its current state, the deployment of two KUKA arms can be considered too extensive and the possibilities for miniaturization must be examined. The MERODES architecture inherently supports the exchange of robots and tools represented in the SDC standard (see Figure 2), easing the reengineering process to incorporate smaller manipulators or decrease their number. The promotion of standardized information exchange among manufacturers of medical robotic systems (e.g., *via* SDC) is crucial in this process. Implementing SDC communication for the KUKA LBR iiwa allows adapting the presented setup for other use cases. The introduction of additional, exchangeable workflow applications facilitates improvements for more challenging puncture interventions in cancer therapy such as US guided cryoablation or non-invasive treatment with high-intensity focused ultrasound (Kim et al., 2019; Ward et al., 2019; Guo et al., 2021).

With its flexible extension possibilities, the presented system provides an addition to the current state of the art of robot-assisted percutaneous interventions. Additional treatment principles can be integrated and the system configuration can be adjusted without much overhead. In future works, the possibilities of interchanging robotic devices and extending the usability of utilized robots in different use cases must be verified. The benefits of standardized device communication in robotic applications must be further investigated. Currently, only a base set of devices (OR-tables, microscopes, etc.) are included in the SDC device network and real-time capability for full robotic integration still poses challenges (Vossel et al., 2017; Schleer et al., 2019).

## Data Availability

The data presented in the study are deposited in the “Frontiers MERODES Data” repository, accessable under https://git.iccas.de/iccas-public/frontiers-merodes-data. Further inquiries can be directed to the corresponding author.

## Footnotes

1 ROS: Home Available at: https://www.ros.org/ [Accessed January 4, 2022].

2 The Orocos Project Available at: https://orocos.org/ [Accessed January 4, 2022].

3 IFL-CAMP/iiwa_stack Available at: https://github.com/IFL-CAMP/iiwa_stack [Accessed January 17, 2022].

4 tf2 - ROS Wiki Available at: http://wiki.ros.org/tf2 [Accessed January 17, 2022].

5 Qt5 (2022). Qt Available at: https://github.com/qt/qt5 [Accessed January 24, 2022].

6 Surgitaix (2021). SDCLib. Available at: https://github.com/surgitaix/sdclib [Accessed January 23, 2022].

7 Clarius Cast API (2022). Clarius Mobile Health - Developer Portal Available at: https://github.com/clariusdev/cast [Accessed January 24, 2022].

8 High Frequency Acoustic Absorber Precision Acoustics. Available at: https://www.acoustics.co.uk/product/aptflex-f28/ [Accessed January 26, 2022].

9 Triple Modality 3D Abdominal Phantom CIRS. Available at: https://www.cirsinc.com/products/ultrasound/zerdine-hydrogel/triple-modality-3d-abdominal-phantom/ [Accessed January 26, 2022].
